# Extract of *Azadirachta indica* (Neem) Leaf Induces Apoptosis in 4T1 Breast Cancer BALB/c Mice

**Published:** 2011-08-24

**Authors:** Fauziah Othman, Gholamreza Motalleb, Sally Lam Tsuey Peng, Asmah Rahmat, Sharida Fakurazi, Chong Pei Pei

**Affiliations:** 1. Human Anatomy Department, Faculty of Medicine and Health Sciences, University Putra Malaysia, Serdang, Selangor, Malaysia; 2. Biology Department, Faculty of Science, University of Zabol, Zabol, Iran; 3. Nutrition and Dietetic Department, Faculty of Medicine and Health Sciences, University Putra Malaysia, Serdang, Selangor, Malaysia; 4. Pathology Department, Faculty of Medicine and Health Sciences, University Putra Malaysia, Serdang, Selangor, Malaysia; 5. Biomedical Science Department, Faculty of Medicine and Health Sciences, University Putra Malaysia, Serdang, Selangor, Malaysia

**Keywords:** Breast Cancer, Apoptosis, Neem, TUNEL

## Abstract

**Objective:**

*Azadirachta indica* (Neem) has been used traditionally for many centuries. Some impressive therapeutic qualities have been discovered. However, the therapeutic effect of neem leaf extract in 4T1 breast cancer has not been documented. The purpose of the present study is to investigate the therapeutic effect of ethanolic Neem leaf extract in an *in vivo* 4T1 breast cancer model in mice.

**Materials and Methods:**

A total of 84 female BALB/c mice were divided randomly into 7 groups (3 non-cancerous groups and 4 cancerous groups) consisting of 12 mice per group. The 3 non-cancerous groups were normal mice treated with 0.5% of Tween 20 in phosphate buffer saline (PBS) (NC), 250 mg/kg Neem (N250) or 500 mg/kg Neem (N500). The 4 cancerous groups were; cancer controls treated with 0.5% of Tween 20 in PBS (CC), and cancerous mice treated with 0.5 µg/mL tamoxifen citrate (CT), 250 mg/kg Neem leaf extract (CN 250) or 500 mg/kg Neem leaf extract (CN 500). Terminal deoxynucleotidyl transferase dUTP nick end labeling (TUNEL) assays were used to evaluate apoptosis (cell death) in the breast cancer tissues. SPSS software, version 14 was used for statistical analysis. Statistical significance was defined as p≤0.05. Non parametric analysis of variance (ANOVA) was performed with the Kruskal Wallis test for the TUNEL assays. Parametric data among the groups was compared using ANOVA.

**Results:**

TUNEL assays showed that the CN 250 and CN 500 groups had a higher incidence of apoptosis compared with the cancer controls.

**Conclusion:**

The findings showed that neem leaf extract induces apoptosis in 4T1 breast cancer BALB/c mice.

## introduction

Despite recent advances in treatment, an estimated 192,370 new cases of invasive breast cancer are expected to occur among women in the US during 2009 and about 1,910 new cases are expected in men (1). Adjuvant chemo- and hormonal therapy have been shown to improve survival in breast cancer patients, but have potentially serious side-effects and are costly. Traditional prognostic and predictive factors are not accurate enough. To improve the indication for adjuvant therapy, additional predictive and prognostic factors are therefore required. Many of them are directly or indirectly related to proliferation or apoptosis (2). Apoptosis is a genetically controlled process and some mechanistic aspects of apoptosis are at least partially conserved throughout evolution. The basic machinery to carry out apoptosis is (constitutively) present in all mammals; however, activation of the apoptotic process is thought to be regulated by the balance between many survival and death signals (3). In many cell types, apoptosis is characterized by the generation of DNA fragments through the action of endogenous endonucleases (4). A large number of biochemical and other tests now exist to study the process of apoptosis in cultured cells, including assays based on morphologi- cal changes, oligonucleosomal DNA fragmentation, cell membrane phospholipid distribution, caspase activation, mitochondrial transmembrane potential and many others. However, there has been a clear need among researchers for methods capable of detecting apoptosis in intact tissue specimens (5). Gavrieli et al. (6) described a procedure for the detection of DNA fragmentation *in situ*, by end labeling of DNA strand breaks using terminal deoxyribonucleotidyl transferase (TdT). Since double-stranded DNA breaks occur during apoptosis, TdT would be expected to preferentially interact with such cells. The dead end fluorometric TUNEL System is designed for the specific detection and quantitation of apoptotic cells within a cell population. The system can be used to assay apoptotic cell death in many systems, including cultured cells and formalin fixed paraffin-embedded tissue sections (6). *Azadirachta indica* (Neem) is known historically for its miraculous healing properties and has been described an ancient cure for a modern world. Neem is the Chinaberry’s miraculous cousin and known as a tree for solving global problems, an epithet which has been recognized by the US National Acedemy of Sciences (7). This plant belongs to the Meliaceae family and is native to the dry forests of India, Pakistan and Sri Lanka. The Malay name is Semambu. The Neem tree’s reputation as a medicinal herb can be traced as far back as 4500 years ago and Neem has been used traditionally for many centuries. Some impressive therapeutic qualities have been discovered; anti-viral, anti-microbial, anti-inflammatory, anti-bacterial, anti-fungal and anti-hyperglycemic. However the anticancer effect of ethanolic Neem leaf extract against breast cancer has not been documented. Previous *in vitro* studies have proved that Neem leaf extract has the potential to induce apoptosis in breast cancer cell lines (MDA-MD231 and MCF-10) (8-10). In the present study, the potential therapeutic effect of ethanolic neem leaf extract in breast cancer was evaluated using the 4T1 breast cancer model in BALB/c mice.

## Materials and Methods

### Cell culture

 Mouse mammary tumour cells (4T1) were purchased from the American type culture collection (ATCC) (Cat. no. CRL2539). All culture work was performed under strict aseptic conditions. The cells were cultured in 10% RPMI-1640 (R1383, Sigma, St Louis, Mo, USA) cell culture media supplemented with 10% fetal calf serum (FCS) (Sigma Aldrich, USA) and 1% penicillin/streptomycin (Sigma Aldrich, USA) in a humidified incubator supplied with 5% CO_2_ at 37℃. The cells were counted using a hemocytometer (Hawksley, England) according to the method of Freshney (11).

### Ethanolic neem leaf extraction

Neem leaf was collected from the UPM area in Selangor in Malaysia. The leaf was identified by Mr Tajuddin Abdul Manap, Agriculture Assistant in the Laboratory of Natural Products, Institute of Bioscience, University Putra Malaysia. Neem leaf extract was prepared as has been described earlier (12). In brief, Neem leaf was left to dry naturally. Dried leaf was then ground to produce a fine powder. 100 grams of powdered Neem leaf was transferred into a borosilicate glass bottle and 200 ml of 80% ethanol was added. The contents were mixed and kept overnight at room temperature. The next day the mixture was filtered into a beaker leaving the residue in the borosilicate glass bottle. Another 200 ml of 80% ethanol was then poured into the borosilicate glass bottle to soak the remaining residue. Again it was kept at room temperature overnight. These steps were repeated for another three consecutive days. Ethanolic extract was evaporated using a rotary evaporator (Rotavapor R-300 BUCHI, Switzerland) at 55℃. Further drying was done using a freeze-drying system (FreeZone 77520, LABCONCO, USA) at -80ºC for 24 hours, after which the extract was dried for another 48 hours in the oven. The extract was then stored at 4℃.

### Animals 

3-4 week old female BALB/c mice were purchased from the Institute of Medical Research (IMR) in Kuala Lumpur in Malaysia and were ethically approved by the Animal Care and Use Committee (ACUC), Faculty of Medicine and Health Sciences, Universiti Putra Malaysia. The mice were housed six to a cage at room temperature with a 12 hours light/12 hours dark schedule and fed autoclaved and water ad-libitum.

### Breast cancer induction

Tumor development was carried out according to the modified method of Xanthopoulos et al. (13). A total of 84 female BALB/c mice were divided randomly into 7 groups (3 normal [non-cancerous] groups and 4 cancerous groups) consisting of 12 mice per group. The 3 non-cancerous groups were normal mice treated with 0.5% of Tween 20 in PBS (NC), normal mice treated with 250 mg/kg neem (N 250) and normal mice treated with 500 mg/kg neem (N 500). The 4 groups of cancerous mice were cancer controls treated with 0.5% Tween 20 in PBS group (CC), cancer treated with 0.5 µg/ml tamoxifen citrate (Sigma, USA) (CT), cancer treated with 250 mg/kg neem (CN 250) and cancer treated with 500 mg/kg neem (CN 500) respectively with intratumoral injections of 0.1 ml of the extract every 48 hours. The mice were injected subcutaneously with 104 4T1 cells suspended in 0.1ml of 10% RPMI-1640 in the region of the left breast. The mice were then monitored and observed for 1 week and the tumors detected by palpation of the induction area. The day the tumor was detected was designated day zero. The tumor was measured every two days with a digital micro caliper (Mitutoyo, Japan) and the size (height, length, width) of the tumor was recorded. The tumor volume was then calculated according to Kotoh et al. (14) as shown below: height (mm) × length (mm) × width (mm). The treatment was continued for 4 weeks after the tumor had developed. Sample collection was done weekly after the treatment started and continued for 4 weeks. At each sampling, 3 mice from each group were humanely sacrificed with diethyl ether and the tumor volume was recorded.

### TUNEL assay

Formalin-fixed paraffin-embedded (FFPE) tumor tissue was used for the TUNEL assay. Briefly, tissues were trimmed and cut by microtome (Leica RM 2135, Germany) at 5µm and pasted onto poly- L-lysine-coated slides (Menzel Glaser, Germany). Tissue sections were deparaffinized by immersing the slides in fresh xylene (I, II) for 5 minutes at room temperature. The slides were then immersed in 100% ethanol for 5 minutes at room temperature and rehydrated by sequential immersing through graded ethanol washes (100%, 95%, 85%, 70%, 50%) for 3 minutes each at room temperature. The samples were then washed by immersing the slides in 0.85% NaCl (5 minutes) followed by PBS (5 minutes). Tissue sections were fixed by immersing the slides in 4% methanol-free formaldehyde solution in PBS (15 minutes). Next, the slides were washed in PBS (5 minutes), a procedure repeated 2 times. 100 µl proteinase K (20 µg/ml) was added to each slide to cover the tissue section. The slides were incubated (10 minutes) at room temperature, then washed in PBS (5 minutes). Tissue sections were fixed in 4% methanol free formaldehyde solution in PBS (5 minutes) and washed again in PBS (5 minutes). The positive control was prepared by treating the slide with DNase I (DNA fragmentation). 100 µl DNase I was added to the section and incubated (5 minutes at room temperature). The liquid was tapped off and 100µl DNase I buffer (10 unit/ml DNase I, Cat. M1601, RQ1 DNase) was added and incubated for 10 minutes at room temperature. Slides were then washed 3 to 4 times in deionized water as a positive control. The positive control was processed in an identical manner for apoptosis detection using separate coplin jars. It is important to use a separate coplin jar for positive control slides. Residual DNase I activity from the positive control slide may introduce high background to the experimental slides. For negative controls, a buffer was prepared without rTdT enzyme (45µl equilibration buffer, 5µl nucleotide mix, and 1µl autoclaved deionized water). The negative control was prepared as follows. Tissue sections were covered with 100 µl equilibration buffer (10 minutes). Nucleotide mix was thawed on ice and sufficient rTdT incubation buffer was prepared for all reactions according to Table 1. Starting from this step on, all following steps were carried out light protected. The equilibrated areas were blotted with tissue paper and 50µl of rTdT incubation buffer was added. Tissue sections were covered with plastic cover slips and the slides incubated (37℃, 60 minutes) inside a humidified chamber. A standard coplin jar was filled with 20× saline-sodium citrate (SSC) was diluted 1:10 with deionized water. After an hour, the cover slips were removed from the humidified chamber and reactions were terminated by immersing the slides in 20× SSC (15 minutes) in the coplin jar. Then, slides were then washed in PBS (5 minutes). This step was repeated 2 times to remove unincorporated fluorescein-12-dUTP. The sections were stained in propidium iodide solution diluted to 1µg/ ml in PBS (15 minutes), then washed in deionized water (5 minutes). This step was repeated 2 times. Afterwards, one drop of anti-fading solution (Biorad) was added to the section and the slides were mounted using glass cover slips. Slides were analyzed immediately under a confocal laser scanning microscope (CLSM) (Biorad, United Kingdom) to view the green fluorescence of fluorescein (520nm ± 20) and view the red fluorescence of propidium iodide (PI) (>620nm). Slides were interpreted for apoptotic TUNEL labeling according to Xu et al. (15). Results are presented as 0: no positively stained cells, 1+: less than 25% slightly positively stained cells; 2+: 25%-50% positive cells; 3+: 50%- 75% positive cells; 4+: >75% positive cells.

### Statistical analysis

Data were expressed as means ± standard deviations. Analysis of variance (ANOVA) was used to compare the means for tumor mass and volume as described earlier (16). Non parametric Analysis of variance (ANOVA) was performed with Kruskal Wallis test for the TUNEL assay (15). A level of p≤0.05 was considered as statistically significant. SPSS software, version 14 (Illinois,USA) was used for the statistical analysis.

## Results

All of the normal mice survived well and completed the 4-weeks treatment. Nevertheless, the mean survival time (MST) of mice in the cancer treated groups was compared with that of the mice in cancer control group (CC). Table 1 shows the results for MST and the percentage increase in lifespan among the Neem treated groups.

**Table 1:** MST and percentage of increase in lifespan in experimental groups under effect of Neem


Group	MST (days) ± Standard deviation	Increase in lifespan (%)
NC	28.00 ± 0.00	Nil
NN 250	28.00 ± 0.00	Nil
NN 500	28.00 ± 0.00	Nil
CC	18.67 ± 0.58	Control
CN 250	20.67 ± 2.08	10.71 %
CN 500	27.00 ± 1.00	44.62 %
CT	28.00 ± 0.00	49.97 %


MST: Mean survival time

**Fig 1:**
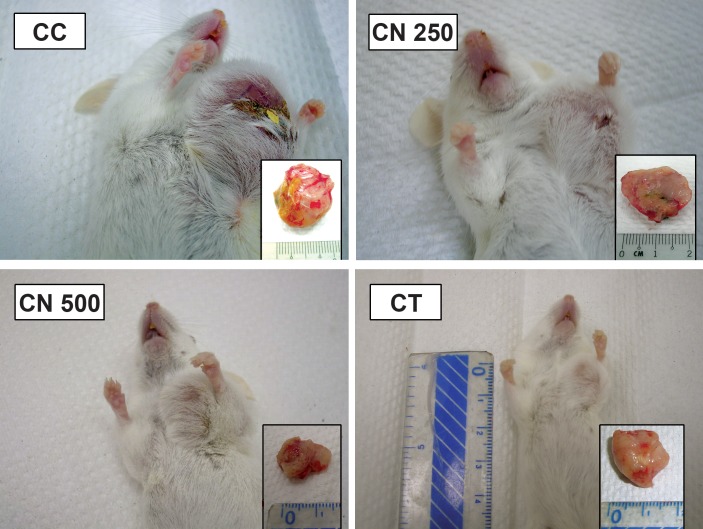
Appearance of 4T1 cells induced breast cancer tumour in Balb/C mice. Note the regression of tumour size in CN 500 group (showed in ruler scale - cm) compared to CC and CN 250 groups

The cancer control mice and the cancer mice treated with 250 mg/kg of Neem (CN 250) survived up to third weeks of treatment. Whereas, cancer mice treated with 500 mg/kg of Neem (CN 500) and tamoxifen citrate (CT) completed the treatment for four weeks. Based on the calculation given by Rajkapoor et al. (17), cancer mice treated with 250 mg/kg of Neem survived up to 20.67 days on average with a 10.71% increase in lifespan. Cancer mice treated with 500 mg/kg of Neem survived up to 27.00 days with a 44.62% increase in lifespan. Mice treated with 0.5 µg/mL tamoxifen citrate, a well established breast cancer drug, survived throughout the period of treatment and had an increase in lifespan of 49.97%. Mean tumour volumes for mice (4T1 breast cancer mouse model) treated with Neem are shown in table 2 and figure 1.

**Fig 2:**
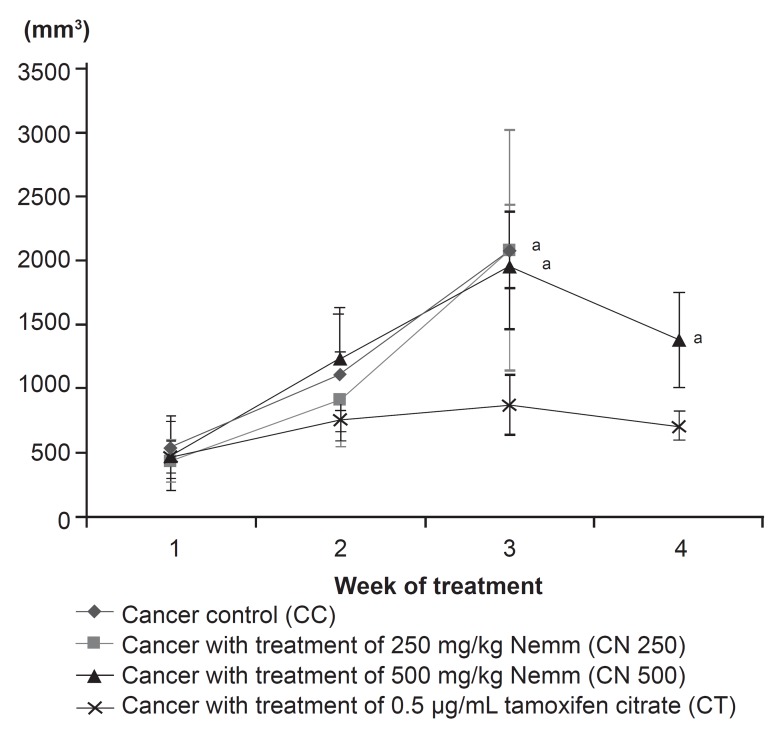
Effect of neem on mean tumour volume changes in the mouse 4T1 breast cancer model. Data are expressed as mean ± standard deviation. a: significant difference with CT group at level of p<0.05

**Table 2:** Mean tumor volume in mice with breast cancer (4T1 breast cancer model) treated with Neem


Group	Mean tumor volume ± Standard deviation (mm^3^)			
	Sampling 1	Sampling 2	Sampling 3	Sampling 4
CC	545.5 ± 241.13	1118.63 ± 451.22	2084.75 ± 298.26	Nil
CN 250	440.29 ± 165.59	914.05 ± 364.65	2079.75 ± 937.40	Nil
CN 500	479.80 ± 273.06	1238.26 ± 400.71	1955.19 ± 483.63	1382.62 ± 367.24
CT	465.50 ± 124.12	759.37 ± 167.19	880.14 ± 236.09	709.95 ± 107.95


MST: Mean survival time

**Fig 3:**
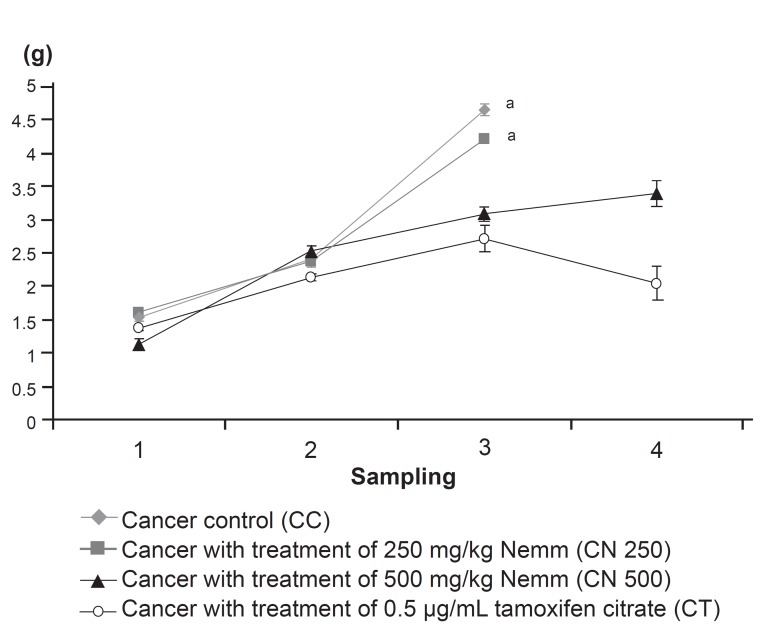
The effect of neem on mean tumour mass changes in the mouse 4T1 breast cancer model. Data are expressed as mean ± standard deviation. a:significant difference with CT group at level of p<0.05

**Table 3:** Mean tumor mass of 4T1 breast cancer model treated with Neem


Group	Mean tumor mass ± Standard deviation (g)			
	S1	S2	S3	S4
CC	1.53 ± 0.05	2.41 ± 0.07	4.65 ± 0.09	Nil
CN 250	1.61 ± 0.06	2.37 ± 0.08	4.20 ± 0.05	Nil
CN 500	1.13 ± 0.08	2.54 ± 0.07	3.08 ± 0.10	3.39 ± 0.18
CT	1.37 ± 0.03	2.13 ± 0.05	2.72 ± 0.20	2.05 ± 0.25


**Fig 4:**
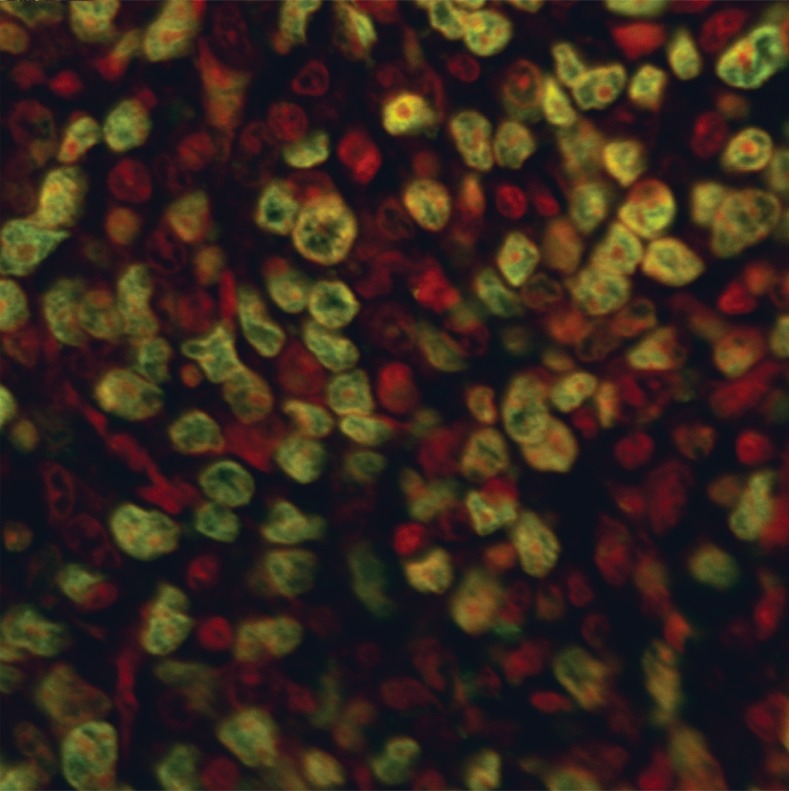
**TUNEL** labeling of the positive control for 4T1 breast cancer tissue pretreated with DNase 1 (Magnification ×400)

**Fig 5:**
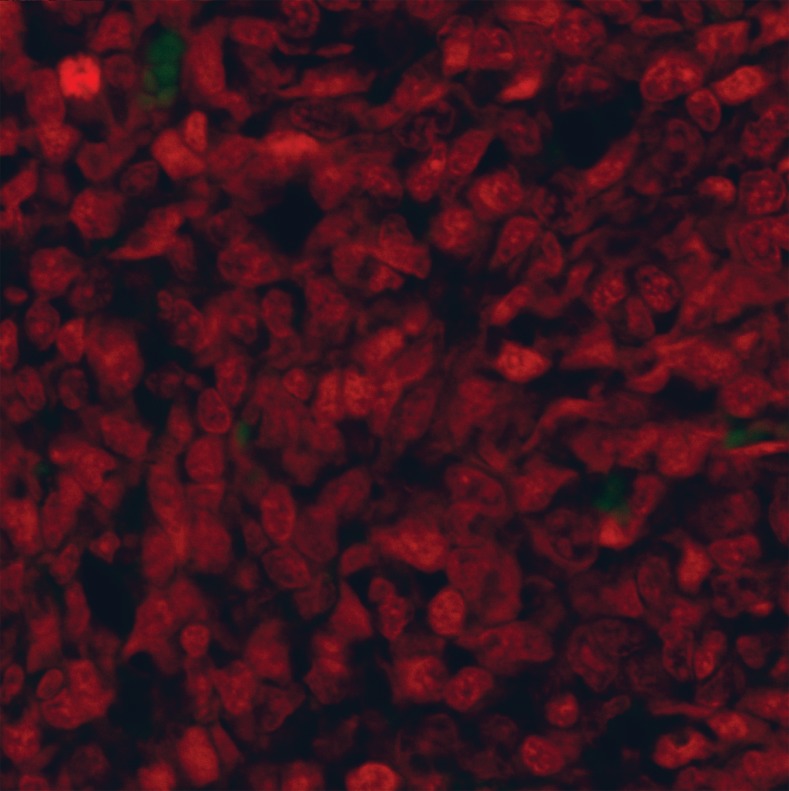
**TUNEL** labeling of the negative control for 4T1 breast cancer tissue without rTdT enzyme (Magnification ×400)

The tumor volumes for mice treated with tamoxifen citrate (CT) were significantly lower compared with mice in the CC, CN 250 and CN 500 groups. However, among these three groups (CC, CN 250 and CN 500), there was no significant difference (p>0.05) in change in tumor volume. Mean tumour mass for the mice treated with Neem are shown in figure 3. Significantly lower mean tumor mass was observed in the CT group compared with the CC and CN 250 group, but there was no significant difference compared with the CN 500 group. The mean tumour mass of the CN 250 group was not significantly different compared with the CN 500 group. Positive control and negative controls were run for each reaction of the TUNEL assay (Figs 4, 5).

**Fig 6:**
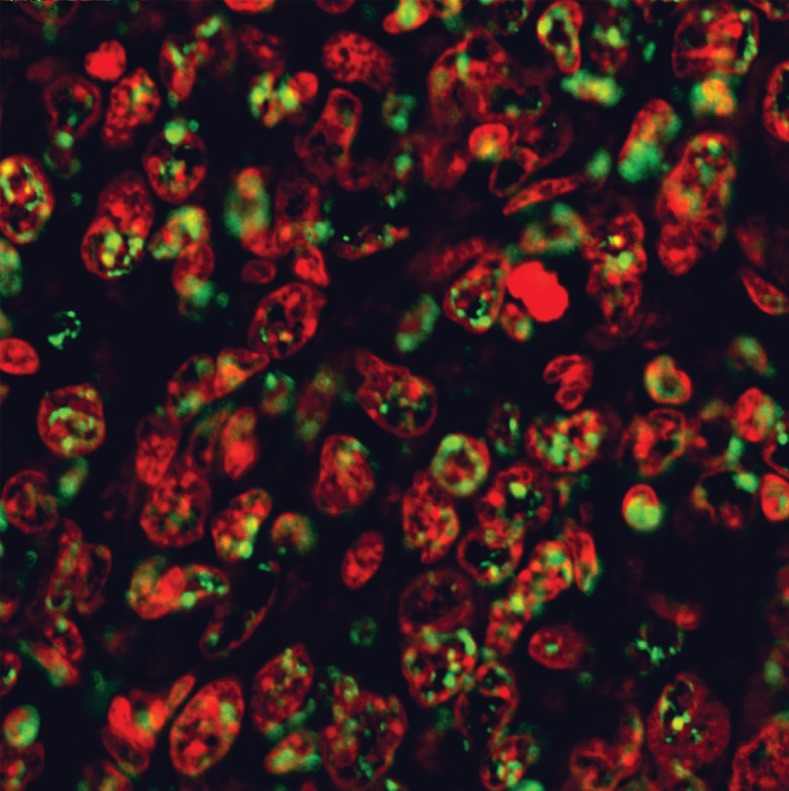
**TUNEL** labeling of breast cancer tissue treated with 250 mg/kg of Neem. Note the small green fluorescent stained dots among the chromosome clots in the nucleus overlapping the PI red fluorescent staining of the DNA nucleus (Magnification ×600)

**Fig 7:**
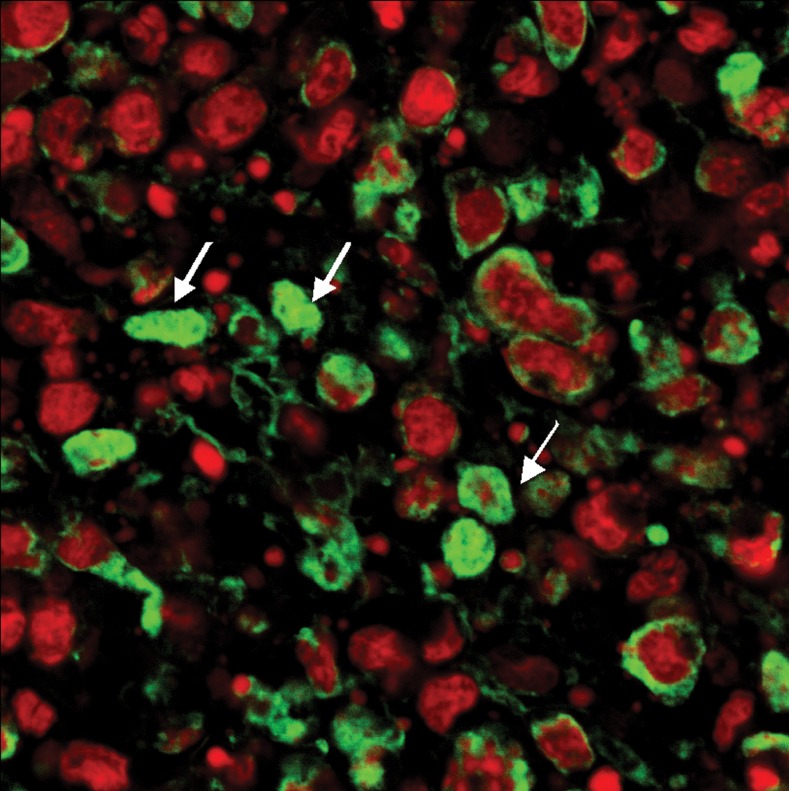
**TUNEL**labeling of cancer tissue treated with 500 mg/kg of Neem. Note some of the cells have dense green fluorescent staining (arrow) (Magnification ×600).

**Fig 8:**
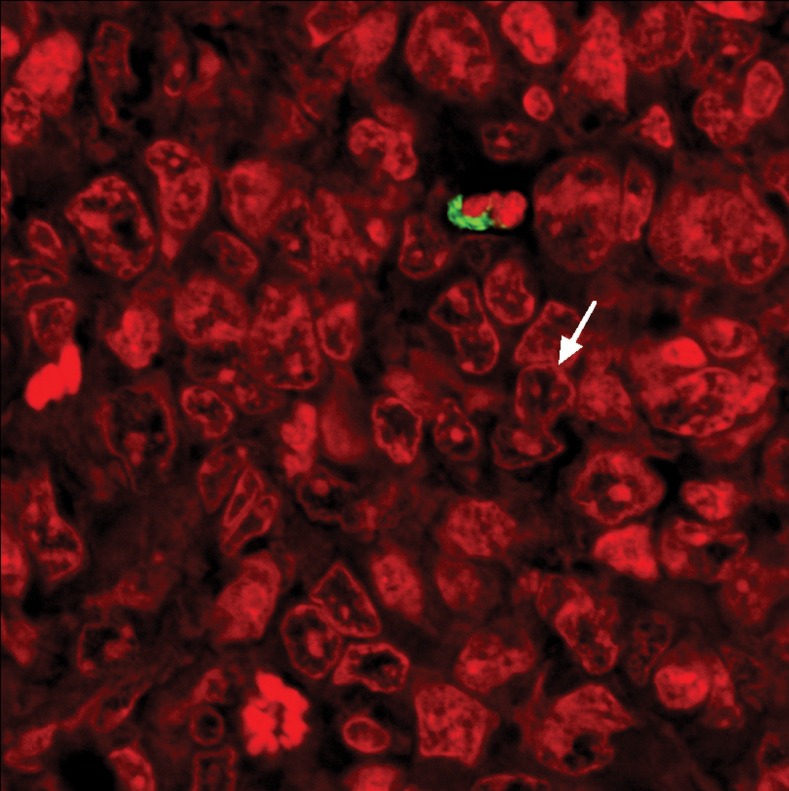
**TUNEL**labeling of cancer tissue in the control group. Note the propidium iodide staining was dominant on the section. A mitotic figure in anaphase (arrow) was observed (Magnification ×600)

**Fig 9:**
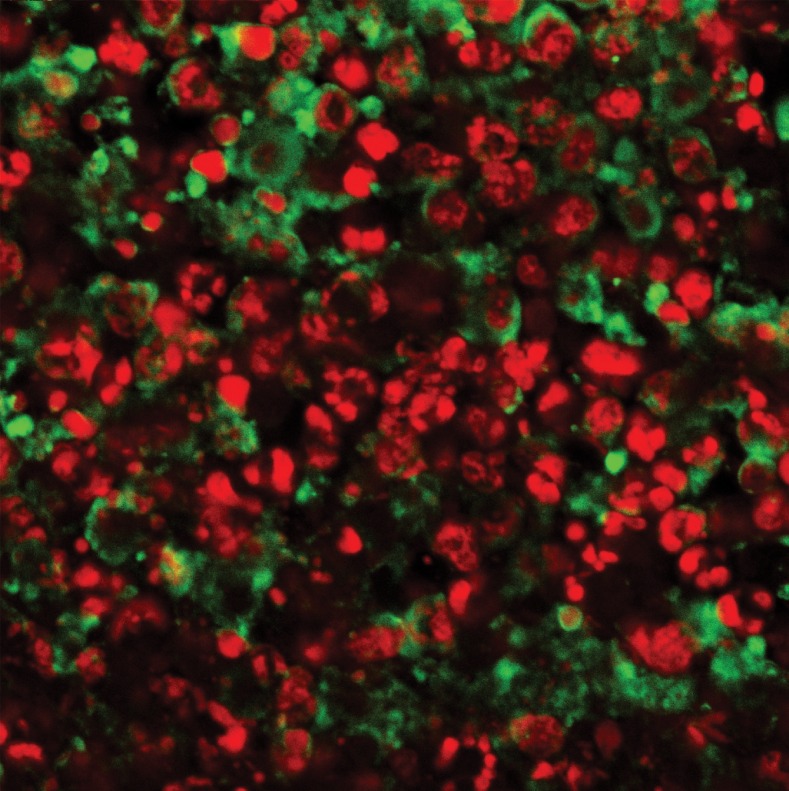
**TUNEL**labeling of cancer tissue in the tamoxifen citrate treatment group. Note the nuclei have stained dense red and green fluorescent staining (Magnification ×600).

In the CN 250, CN 500, CC and CT groups, differential green and red fluorescent staining indicated the behavior of the nuclei (Figs 6-9).

**Fig 10:**
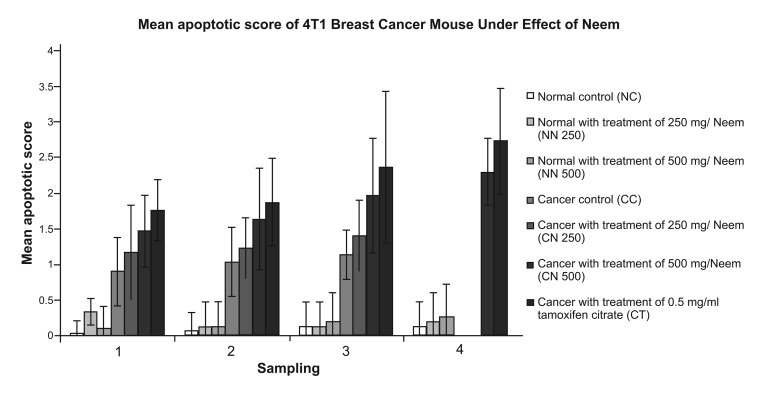
Mean apoptotic score of 4T1 breast cancer mouse model under effect of Neem. Data are expressed as mean ± standard deviation. a: significant difference with NC, NN 250 and NN 500 group at level of p < 0.05. b: significant difference with CC group at level of p < 0.05.c: significant difference with CN 250 group at level of p < 0.05. d: significant difference with CN 500 group at level of p < 0.05

The number of apoptotic cells in the CN 500 group was significantly higher than in the CC and CN 250 groups whereas, there was no significant difference compared with the CT group (Fig 10). As in the CN 250 group (Fig 6), the nucleus in the plane of view was not as crowded as in the CC samples. There were small dots of green fluorescent staining among the chromosome clots in the nucleus overlapping the PI red fluorescent staining of the DNA in the nucleus. Interestingly in the CN 500 group (Fig 7), the majority of the cells had condensed nuclei with intense red fluorescent staining. Concurrently, there were also a number of condensed nuclei stained with intense apoptotic green fluorescent staining which were never observed in the CN 250 samples. This green stain was found in the condensed nuclei as well as around the nuclear membrane. Some of the nuclei of the cancer cells were breaking apart into small pieces in contrast to the prominent nuclei found in the CC samples. In the CC group, the PI stain was dominant however, the appearance of the stained nucleus was swollen and prominent and many dark clots of chromosomes were observed with the presence of hairy extensions when focusing up and down (Fig 8). In the CT group, the appearance of the red staining (Fig 9) revealed that the nuclei of the cells had broken apart into small pieces and in a number the background staining of the nucleus was green instead of red. The quantitative data for apoptotic cells are presented in Fig 10. The CT group exhibited the highest percentage of apoptotic cells, ranging from 44.2 to 68.3%. In comparison, the number of apoptotic cells in the CN 500 group was significantly higher than in the CC group. Both the CN 250 and CN 500 groups showed an increase in apoptotic cells, ranging from 29.2 to 35.0% and 36.7 % to 57.5 % respectively throughout the experiment.

## Discussion

The 4T1 breast cancer mouse model was used *in vivo* to evaluate the efficacy of ethanolic Neem leaf extract in the present study.

model for this type of study because the mammary glands of mice and humans are very similar in structure and function (18). Fauziah et al. (19) successfully used TUNEL assays to detect apoptotic cells in the MCF-7 breast cancer cell line. We applied the terminal deoxynucleotidyl transferase dUTP nick end labeling (TUNEL) assay to evaluate apoptosis in breast cancer tissues by confocal laser scanning microscopy. The treatment doses of Neem given to the mice in this study were considered as low compared to its LD50 value, described previously (20). In other words, 250 and 500mg/kg concentrations of Neem are safe for mice. Interestingly, mice in the CC and CN 250 groups survived until week 3, whereas mice in the CN 500 and CT groups were able to survive till the end of the experiment. From the results obtained, treatment with 500mg/kg of Neem significantly increased survival time in mice (4T1 breast cancer model) by as much as 44.62%; providing strong support for its effectiveness in treating the breast cancer in mice (4T1 breast cancer model). It was also quite similar to tamoxifen citrate treatment in terms of survival rate (49.97%). However, treatment with 250 mg/kg of Neem gave only a 10.71% increase in lifespan compared to the CC group. The concentration of Neem given in the treatment was proportional to its effectiveness against mouse breast cancer (4T1 breast cancer model). These results are similar to those of Sarkar et al. (12) who found that Neem leaf extract significantly increased survival time in a colorectal tumor model in mice whereas, our results were dose dependent. In addition, they showed that Neem leaf extract significantly restricted colorectal tumor growth during the study. Interestingly, in our experiment lower tumor mass and tumor volume was observed in the CN 500 group. However, these were not significantly different compared with the cancer control group (Fig 1). Sections stained with fluorescein-12-dUTP in the absence of rTdT enzyme showed no green background staining, confirming that positive staining in the experimental sections was enzyme mediated. When fluorescein was used as the fluorescent dye in the TUNEL assay, it was discovered that propidium iodide (PI) was suitable for DNA staining, because PI binds to the nucleotide pair guanine and cytosine (21). During TUNEL labeling, the cell membranes were digested by proteinase K to ease penetration of both dyes to react with the nucleic acids found in nuclei. TUNEL systems label the nucleus of the cell. The morphology of the nucleus was highlighted by both fluorescent dyes which enabled us to interpret the TUNEL micrograph both qualitatively and quantitatively. Experience in evaluation is needed to judge and recognize true positive apoptotic cells via their morphological appearance. For this reason it is strongly recommended that at least two methods to measure the different features of the apoptotic process are employed to obtain reproducible results in the detection of apoptosis (22). In this study, apoptosis was evaluated using different levels of microscopy (light and electron microscopy, unpublished data) together with *in situ* apoptotic cell labeling for confirmation of apoptosis. Despite the TUNEL reactivity, cells must show the morphological features of apoptosis (nuclear shrinkage, chromatin condensation, retraction from surrounding cells or apoptotic bodies) in order to confirm the identity of positive cells as apoptotic cells. A green stain was associated with condensed chromatin and the inside of the nuclear membrane. Deposition of green stain in cancer treated cells was therefore in agreement with the respective appearance found using light and electron microscopy (unpublished data). The chromatin in apoptotic cells was clumped and was integrated with the nuclear membrane. Tamoxifen citrate (commonly known as tamoxifen) is a well established drug for the treatment of breast cancer (23). Tamoxifen citrate was observed to suppress tumor growth in the 4T1 breast cancer model, with evidence of apoptotic induction provided by morphological analysis and apoptotic cell staining via TUNEL assay. The apoptotic cells were found in both the CN 250 and CN 500 groups. However, deposition of green stain in the apoptotic nucleus in the CN 250 and CN 500 groups differed from each other, indicating cells were in different stages of apoptosis. In the CN 250 group, a small deposition of green stain in chromatin clots indicated they were in the early stage of apoptosis. However, most cells in the CN 500 group were in the late stage of apoptosis, displaying a condensed nucleus with intense green stain. Some of the nuclei of the cancer cells were breaking apart into small pieces; indicating the presence of apoptotic bodies and a late stage of apoptosis. In the CT group, many apoptotic bodies were found, again indication of a late stage of apoptosis. In addition, green staining and red staining were distributed evenly, proving that tamoxifen citrate was effective against 4T1 cells through inducing apoptosis mechanisms (Fig 9). The findings showed 500mg/ kg of neem is more effective at inducing apoptosis in 4T1 mouse breast cancer cells. Our result is very similar to that of Dessy et al. (24) who found that Neem extract induced apoptosis in MCF-7 breast cancer cell lines. In addition, a few studies have revealed that ethanolic neem leaf extract induces apoptosis in the MDA-MD 231 breast cancer cell line (8, 9). The apoptotic score of the CN 250 group was similar to that of the CC group, whereas the apoptotic score of the CN 500 group was statistically similar to that of the CT group. These findings signified that 500mg/kg of Neem has a greater effect on apoptosis induction against the 4T1 cells. The effectiveness and mechanism of the ethanolic Neem leaf extract against 4T1 breast cancer cells in the mouse model has yet to be elucidated using morphological evaluation by transmission electron microscopy (TEM), and *in situ* RT-PCR breast cancer oncogene expression (unpublished data). Once the efficacy of ethanolic Neem leaf extract as an anti breast cancer agent has been established, its tolerance, toxicity, stage specificity and mechanism of action should be determined to enhance its anti-cancer value. Further study on the expression of more related breast cancer oncogenes will be needed to enhance the findings in this study.

## Conclusion

A dose of 500 mg/kg of ethanolic neem leaf extract was found to induce apoptosis in 4T1 breast cancer cells in a mouse model. Apoptosis was successfully confirmed by TUNEL assay.
